# Profiles and trajectories of impaired social cognition in people with Prader-Willi syndrome

**DOI:** 10.1371/journal.pone.0223162

**Published:** 2019-10-17

**Authors:** Elisabeth M. Dykens, Elizabeth Roof, Hailee Hunt-Hawkins, Christopher Daniell, Sarah Jurgensmeyer

**Affiliations:** 1 Department of Psychology and Human Development, Vanderbilt University, Nashville, TN, United States of America; 2 Vanderbilt Kennedy Center for Research on Human Development, Vanderbilt University Medical Center, Nashville, TN, United States of America; 3 Vanderbilt Kennedy Center University Center of Excellence on Developmental Disabilities, Vanderbilt University Medical Center, Nashville, TN, United States of America; Birkbeck University of London, UNITED KINGDOM

## Abstract

**Introduction:**

People with Prader-Willi syndrome (PWS) have a distinctive behavioral phenotype that includes intellectual disability, compulsivity, inattention, inflexibility and insistence on sameness. Inflexibility and inattention are at odds with the cognitive flexibility and attention to social cues needed to accurately perceive the social world, and implicate problems in social cognition. This study assessed two social cognition domains in people with PWS; emotion recognition and social perception. We identified changes in social cognition over an approximate two-year time period (*M* = 2.23 years), relative strengths and weakness in social cognition, and correlates and predictors of social cognition.

**Methods:**

Emotion recognition and social perception were examined at two time points in 94 individuals with PWS aged 5 to 62 years (*M* = 13.81, *SD* = 10.69). Tasks administered included: standardized IQ testing; parent-completed measures of inattention and inflexibility; standard emotion recognition photos (fear, sadness, anger, happy); and videotaped social perception vignettes depicting negative events with either sincere/benign or insincere/hostile interactions between peers.

**Results:**

An atypical trajectory of negative emotion recognition emerged, marked by similar levels of poor performances across age, and confusion between sad and anger that is typically resolved in early childhood. Recognition of sad and fear were positively correlated with IQ. Participants made gains over time detecting social cues, but not in forming correct conclusions about the intentions of others. Accurately judging sincere intentions remained a significant weakness over time. Relative to sincere intentions, participant’s performed significantly better in detecting negative social cues, and correctly judging trickery, deceit and lying. Age, IQ, inattention, and recognition of happy and sad accounted for 29% of variance in social perception.

**Conclusion:**

Many people with PWS have deficits in recognizing sad, anger and fear, and accurately perceiving the sincere intentions of other people. The impact of these deficits on social behavior and relationships need to be better understood.

## Introduction

People with intellectual disabilities or neurodevelopmental disorders often experience some degree of difficulty in social adaptation or functioning [1]. How these individuals adapt to the demands of family and community life depends, in part, on how they perceive the emotions, intentions, and behaviors of themselves and others, broadly conceptualized as social cognition [2]. Encompassing distinct but interrelated domains, social cognition includes abilities to: take another’s perspective (theory of mind); recognize the emotional states of others; use cues to draw inferences about social situations (social perception); and adopt a personal framework for making causal explanations for social events (attributional style) [3]. Deficits in these domains are defining features of such neurodevelopmental disorders as autism or schizophrenia [4], yet they may also be problematic in other neurodevelopmental disorders, including Prader-Willi syndrome (PWS).

PWS is caused by a lack of paternally derived, imprinted information at 15q11-q13, either through paternal deletions that differ in size (Type I, II and atypical deletions), or less commonly via maternal uniparental disomy (mUPD; when both copies of chromosome 15 are maternally inherited.) [5,6]. People with PWS typically show mild to moderate intellectual disability; growth hormone deficiencies; hyperphagia and high risk for life-threatening obesity; and aggressive behaviors, rigid thinking, irritability, needs for sameness, and compulsivity [7,8].

People with PWS also have significant problems relating to peers, sustaining friendships, and getting along with others [9,10]. They may have specific impairments that deter their abilities to successfully interact with others, or to form meaningful, reciprocal relationships with them. For example, based on the Autism Diagnostic Observation Schedule-2 [11], Dykens and colleagues [12] reported frequent problems in the quality and amount of reciprocal social communication in 146 children and youth with PWS. Even though the majority of participants (87.7%) did not meet criteria for autism spectrum disorder (ASD), they still evidenced sub- threshold impairments in the quality of social overtures, rapport with the examiner, reporting of events, reading cues, and insight [9]. While poor event reporting or insight may relate to cognitive deficits, difficulties reading social cues have a negative, cascading effect on social cognition. Frith and Frith [13] emphasize that reading social signals, including emotions, are the gatekeepers to learning from others and accurately perceiving the intentions of people.

Just two domains of social cognition have been previously studied in PWS—theory of mind and emotion recognition. Administering theory of mind tasks to 66 children and youth with PWS, Lo and colleagues [14] found that participants understood another person’s mistaken belief (first order false-belief), but performed poorly when asked to form inferences of someone’s belief about another’s belief (second-order belief). Performances on these tasks were associated with Verbal IQ, but not with age or PWS genetic subtype.

People with PWS also have difficulties recognizing emotional cues within social contexts. Koening et al. [15] administered a short video of moving shapes in which 18 adolescents with PWS had to first recognize the visual stimuli as social phenomena, and then extract cues from the video to create a meaningful story. Participants with PWS performed worse than IQ matched controls, and on par with those with pervasive developmental disorders. It was particularly difficult for those with PWS to ascribe affective states to the moving stimuli, which reduced the quality of their social stories.

Identifying affective states from facial expressions is also problematic for many with PWS. Among 52 children and adults with PWS, Whittington and Holland [16] report that while most could recognize happiness, fewer could correctly identify negative emotions (e.g., sad, angry, worried). Adults with histories of depression were more impaired in recognizing fear, and those with psychosis in recognizing anger. Emotion recognition was associated with IQ, but not with age or PWS genetic subtype.

The present study addressed several salient gaps in the PWS social cognition literature. First, researchers have yet to explore the social perceptions of those with PWS, or how they use cues to draw inferences about social situations. Social perception is often measured via scenarios in which respondents must use verbal and/or nonverbal cues to interpret ambiguous or conflictual social situations [17]. Accurately interpreting social scenarios depends on several executive function skills, including attending to pertinent social cues and being cognitively flexible in order to change ones’ ideas or behaviors in response to social stimuli [18]. People with PWS, however, typically exhibit: rigid thinking; inflexibility; needs for sameness; resistance to change; inattention and impulsivity [7,8,9,19]. Woodcock and colleagues [19] identified specific deficits in task switching in 28 children with PWS that predicted their preferences for routine, repetitive questioning, and temper outbursts. Further, symptoms or diagnoses of Attention Deficit Hyperactivity Disorder (ADHD) have been reported in up to 70% of children with PWS [20,21], as have problems with selective and divided attention [19]. Both inattention and inflexibility may thus impede the ability of people with PWS to attend to and process divergent social cues, which may ultimately contribute to faulty social perceptions.

A second research gap concerns changes over time in social cognition. It is well established that both emotion recognition and social perception skills develop across infancy, childhood and adolescence [22]. While basic social cognition skills develop throughout childhood (e.g., recognizing straightforward emotions, theory of mind), more complex skills emerge in adolescence and young adulthood, including recognizing complicated emotions (e.g., sexual/romantic interest, fear, contempt) or social perception skills (e.g., detecting white lies, irony, dares) [23,24,25]. Indeed, relative to children or adolescents, adults perform better on these more complex tasks, underscoring that social cognition abilities evolve from basic to more nuanced understandings of emotional expressions and social exchanges over time.

Even so, the trajectories of social cognition are understudied in people with intellectual disabilities in general [2], including those with PWS. Given their intellectual disabilities, people with PWS may show delays in social cognition skill acquisition, yet still follow a similar developmental course as the typically developing population. This possibility reflects the theoretical assumption that people with intellectual disabilities go through the same sequences of development as typically developing individuals, but at a slower rate [26]. Alternatively, cognitive impairments or other factors (e.g., hyperphagia, compulsions) may impose constraints on social cognitive skills, leading to an atypical developmental trajectory. Theoretically, this possibility presumes that those with intellectual disabilities have core deficits or features that set the apart from others and lead to altered developmental courses [27].

Finally, social cognition theorists increasingly appreciate that the ability to recognize emotions in oneself and others helps people accurately decipher social interactions. Lemerise and Arsenio [28] assert that while people bring their own emotional arousal or mood to social interactions, they also use the emotional cues of others to guide how they encode and interpret social situations. Similarly, Ladd and Crick [29] propose that emotions play an important role in social information processing, especially evaluating one’s own responses to social interactions. Those who struggle to recognize basic emotions in others may thus be at a disadvantage in forming accurate perceptions of social situations.

In brief, the present study examined how 94 participants with PWS performed on two domains of social cognition—emotion recognition and social perception—assessed at two different time points. As the development of these skills is relatively under studied in people with intellectual disabilities, we were uncertain if people with PWS would show relative stability, gains, or atypical developmental patterns in emotion recognition or social perception skills. We thus first examined changes over time for the sample as a whole, and the effects of age on these changes. Second, we assessed relative strengths and weaknesses in emotion recognition and social perception. Based on previous literature [16], we expected to find strengths in recognizing happy relative to negative emotions. On the social perception task, we predicted that participants with PWS would perform better when provided with obvious or blatant cues (e.g., a sincere apology) as opposed to subtle, harder to read social cues (e.g., a sarcastic remark). Finally, we predicted that lower cognition and heightened inattention and/or inflexibility would detract from participant’s performances. Controlling for these potential predictors, and consistent with the role of emotions in forming social percepts, we expected that basic emotion recognition skills would account for some of the variability in social perception task performance.

## Methods and materials

### Participants

The sample included 94 children, adolescents, and adults aged 5 to 62 years with genetically confirmed PWS (45.7% male, 54.3% female). Longitudinal power analyses using standard parameters, alpha < .05, power = 80%, two-sided tests, and an effect size of 0.5, yielded a sample size of 63 [30]. The study was thus appropriately powered to detect medium to small effect sizes.

Families were recruited from throughout the U.S. to participate in a longitudinal study on behavior and development in PWS. Given their wide age range, participants were divided into three age groups: children aged 5 to 10 years (n = 44); adolescents aged 11 to 19 years (n = 34); and adults aged 20 to 52 (n = 16); see Table 1 for the mean ages of these groups. These are developmentally appropriate age groups, and as previously discussed, they also index periods of growth (children, adolescents and young adults) or stability (adults) in social cognition skills in the general population. The distribution of PWS genetic subtypes, also noted in Table 1, did not differ by age groups. Overall, 61.7% had paternal deletions, 29.8% mUPD, and 8.5% other, more rare genetic subtypes. K-BIT2 Composite IQ scores of the sample ranged from 40 to 130, *M* = 70.71, *SD* = 17.28; 48.5% had IQ scores of 70 or higher. As Table 1 indicates, children had higher IQ scores than remaining groups, *F*(2,193) = 14.47, *p*< .001. This trend, consistent with previous studies [31], likely relates to cohort effects and the cognitive benefits of growth hormone treatment (GHT) [32], which is FDA approved for children and youth. As such, the effect of GHT status on task performance was examined, and IQ was controlled for in between age-group analyses.

**Table 1:** Participant demographics and behavioral characteristics across age groups.

	5 to 10 years	11 to 19 years	20 years and up	Total or Overall *M*
N	44	34	16	94
*M* Age Time 1	6.31 (1.44)	13.97 (2.52)	33.58 (12.02)	13.64 (10.83)
*M* Age Time 2	8.72 (1.82)	16.14 (2.64)	35.70 (12.11)	15.95 (10.81)
*M* Test Retest Interval, years	2.29 (.60)	2.23 (.54)	2.03 (.59)	2.23 (.58)
Gender	47.7% M	50.0% M	41.3% M	45.7% M
*M* Verbal IQ	84.53 (14.02)	75.79 (14.55)	70.04 (14.11)	76.78 (14.19)
*M* Nonverbal IQ	78.31 (17.01)	66.33 (18.76)	64.96 (18.49)	69.87 (18.08)
*M* Composite IQ	78.79 (15.73)	67.47 (16.73)	63.96 (16.41)	70.71 (17.38)
*M* Attention Problems	8.30 (4.02)	7.67 (3.97)	6.12 (3.15)	7.50 (3.87)
*M* Sameness/Rituals	17.32 (7.76)	18.10 (6.64)	16.58 (6.70)	17.36 (7.10)
Genetic Subtypes				
Type I Deletion	18.2%	17.6%	37.5%	21.3%
Type II Deletion	40.9%	41.2%	37.5%	40.4%
mUPD	29.5%	35.3%	18.8%	29.8%
Other*	11.4%	5.9%	6.3%	8.5%

Note

*The Other genetic subtype category included 5 individuals with imprinting center defects, and 3 with unique deletions.

We ensured that the 94 participants understood the expectations of the social cognition tasks and had adequate expressive language and verbal skills to complete the work at hand (see Table 1 for Verbal IQ’s). An additional 4 individuals were excluded from the study as they did not appear to understand the tasks and/or their responses to the test stimuli were highly perseverative.

The test-retest interval ranged from 1.5 to 4 years, and averaged 2.28 years. Table 1 shows the mean test-retest interval across age groups, which were similar across groups. Test-retest intervals were not significantly associated with social cognitive tasks, and as such, were not used as a control variable in analyses.

## Procedures

Prior to enrolling participants, this study was first approved by Vanderbilt University’s IRB Social/Behavioral Sciences Committee. Consistent with University IRB regulations, parents of offspring with PWS provided written, informed consent for the study, and individuals with PWS provided written, informed assent.

Following consent or assent procedures, a test battery was individually administered in a quiet room by trained research assistants who were highly experienced in working with individuals with PWS and their families. The test battery included the following measures.

### Social cognition assessments

#### Emotion recognition

Emotion recognition was assessed with 24 standardized photos from the Japanese and Caucasian Facial Expressions of Emotion (JACFEE) [33].

Stimuli included 11 Caucasian women and 13 men who depicted six emotions: happy, sad, angry, fear/afraid, disgust and contempt. Each emotion had four photos that were randomly administered using the prompt “I am going to show you pictures and I want you to tell me how that person feels. Are you ready?”

Given their cognitive deficits, no time limits for responding were imposed. Additional prompts were used on an as-needed basis, e.g., “Take another look, how is (s)he feeling?” Responses based on physical attributes of the photo (e.g., “smiling”) were prompted with “How does she feel when she’s smiling?”

#### Emotion recognition scoring

Scores ranged from 0 (none correct) to 4 (all correct) for each emotion. The following responses were scored as correct: Angry = angry, anger, mad, furious, irritated, pissed, grumpy, ticked off; Happy = happy, excited, silly, gleeful; Sad = sad, disappointed, unhappy, depressed, down in the dumps; and Afraid = scared, nervous, anxious, fearful, frightened, worried. Just 2 adults could reliably identify contempt or disgust at Time 1 or Time 2. Given this floor effect, these emotions were not included in analyses.

#### Social perception task

Developed by Leffert and colleagues [34], this task is based theoretically on models of social cognition that distinguish between two related processes; encoding and interpreting social stimuli [35]. It was specifically designed to assess how individuals with intellectual disabilities attend to, encode, and interpret the intentions of others. The task consists of six, brief, 1–2 minute videotaped vignettes using youth actors in a school setting that depict a problematic situation or negative event that places one of the characters at a disadvantage. Participants were instructed to pay special attention to the protagonist (e.g., “the boy with the blue shirt”) because they would be answering questions about him/her and the story.

Three vignettes depicted negative events involving sincere/benign intentions in which either the negative event or the social cues differed in salience. In one, involving a *subtle apology*, two friends are doing their homework together, and as one leaves to get pencils the other student reaches for a clean piece of paper and unknowingly writes on the back of her friend’s homework. Her friend returns and demands, “What are you doing? You scribbled all over the final copy of my book report!” to which the student embarrassingly says “uh oh!” The second, illustrating a *blatant apology*, depicts a student at her locker packing up her backpack. As she swings it on her back, it accidently bumps into a student, causing her to drop her stack of unclipped papers, and exclaim “Oh no! Now they’re all out of order!” to which the girl responds, “I’m so sorry! I should’ve been watching where I was going!” And the third portrays an *excuse with strong emotions*. Two boys plan to see a movie after school, but at the end of the day, one of them remembers that he has a doctor’s appointment. He tells his friend that they can't see the movie, the peer becomes visibly aggravated and cries out “You’re just telling me now?” to which the other boy defensively responds, “I told you I forgot that I had a doctor’s appointment!”

Three scenarios conveyed insincere/hostile intentions, with insincere cues mixed with ostensibly benign cues. In one, depicting *manipulation and rejection*, a student asks to join two friends playing catch, and is told “Sure, but let us finish this game first. Do you mind grabbing a jump rope while you wait?” When she returns with the jump rope, the girl say “Thanks!” takes the jump rope, gives her the ball and skips away with her friend saying” Let’s go play jump rope!” In another involving *teasing and rejection*, a group of girls is making fun of another student’s clumsiness at sports. The student overhears them and says, “It's really not nice to call people names, you know” to which one of the girls sarcastically replies, “Oh, we weren’t making fun of you, you are a really smart kid, and not everyone can be good at sports.” In the third vignette depicting *lying and rejection*, a student approaches a game of jump rope, asks a girl who is swinging one end of the rope if she can play, and is told no, they already have enough players. Another girl approaches, also asks to play and is told, “Sure, you’re next” to which the first girl asks, “How come you let her in and not me?” The student swinging the rope stalls for time, looks up, then away, and without looking at the girl eventually says “Well …um …ah, I already told her that she could play with us. Sorry.”

#### Vignette scoring

At the end of each vignette, participants were asked, “What happened?” and prompted as needed (e.g., “Then what happened” or “Anything else?”). Their responses were recorded and evaluated against a checklist, provided by the test developers, of 7 to 9 pertinent events and social cues imbedded in each story. For example, in the vignette with the dropped papers, the checklist includes 3 items related to the negative event (child A drops papers, papers are out of order, child A is upset) and 4 benign cues (child B didn’t see child A when she swung her bag around; Child B apologizes; Child B adds “I should have ben watching where I was going”; participant makes any mention of hitting the papers by accident). The checklist thus indexes participants’ observations of the presence (or absence) of those social cues that guide the accurate interpretation of the story. As vignettes varied in the number of hostile or benign cues, proportions were calculated for each type of response. The proportions of negative and benign cues were then summed across the 3 sincere and insincere vignettes, respectively.

After recalling the story, participants were then asked to judge whether the protagonist was either “mean” or “not mean”. Responses were scored as correct (i.e., not mean in the sincere scenarios, and mean in the insincere vignettes) or incorrect (mean in the sincere vignettes or not mean in the insincere scenarios).

### Correlates of social cognition

Performances on the two social cognition tasks were examined in relation to: cognition (IQ), inattention, and needs for sameness/inflexibility.

#### Inattention

Inattention was assessed by the Child Behavior Checklist [36], a widely used, 113-item, parent-completed measure of problem behaviors. The CBCL has excellent psychometric properties, and has been used in other studies of individuals with developmental disabilities. The CBCL yields two broad domains (Internalizing, Externalizing problems) and 9 subdomains, including Attention Problems. The Attention Problems subdomain is comprised of 10 items, e.g., “Can’t concentrate”, “Inattentive”, “Impulsive, acts without thinking” and “Fails to finish things (s)he starts”. Given the age range of our sample, one item was modified to be appropriate for both children and adults (poor school/work evaluations). Consistent with prior CBCL studies that include both children and adults with developmental disabilities, Attention Problems raw scores were used in data analyses. Scores in our participants ranged from 1 to 19, with a mean of 7.50, *SD* = 3.87, see Table 1.

#### Cognition

Participants were individually administered the Kaufman Brief Intelligence Test-2 (KBIT-2) [37], which was designed for research and screening purposes. The KBIT-2 has been successfully used in previous studies of people with developmental disabilities. Compared to “short forms” of traditional IQ tests, the KBIT-2 has more robust psychometric properties [38]. The KBIT-2 provides standard scores (*M* = 100, *SD* = 15) for Verbal, Nonverbal and Composite IQ’s. See Table 1 for means scores across age groups.

#### Inflexibility

The Repetitive Behavior Scale-Revised RBS-R [39, 40] was used to measure needs for sameness and inflexibility. The RBS-R assesses a wide range of restricted and repetitive behaviors in people with developmental disabilities. Informants complete 43 items using a four-point Likert scale: 0 = behavior does not occur; 1 = behavior occurs and is a mild problem; 2 = behavior occurs and is a moderate problem; and 3 = behavior occurs and is a severe problem. Data analyses used the 17 items that comprise the Sameness/Rituals factor of the RBS-R. This factor aptly reflects the inflexibility that is highly characteristic of PWS, including: “Resists changing activities”, “Insists on the same routine household, work or school schedules every day”; “Becomes upset if interrupted in what he/she is doing”; and “Repetitive questioning or insisting on certain topics of conversation.” Higher raw scores index more problems. Scores in our sample ranged from 3 to 42, with an overall mean of 17.36, *SD* = 7.10, see Table 1.

### Statistical analytic plan

#### Emotion recognition data analyses

We first conducted four, 2 X 3 ANCOVA’s (time by 3 age groups) for each of the 4 emotion recognition scores, controlling for IQ. ANCOVA’s allowed us to identify main effects between test times for the group as a whole, between age groups, and possible time by age group interactions. Changes within each age group were assessed by matched t-tests comparing Time 1 and Time 2 emotion recognition scores. Collapsing across age and assessment times, relative strengths or weaknesses in recognizing specific emotions were assessed in a within-group, repeated measure ANOVA. Again collapsing across assessment times and age, Pearson correlations were conducted between emotion recognition scores and age, Composite IQs, the CBCL’s Attention Problem subdomain and the RBS-R Sameness/Rituals Domain.

Finally, an error analysis was conducted to determine if participant’s incorrect responses were markedly discrepant from the negative emotions portrayed in the facial stimuli. Incorrect responses were reviewed and categorized as: happy or another positive affective state (e.g., “proud”, “courageous”); one of the other emotions under study; or a more general negative statement (e.g., “grossed out”, “confused”, “tired”, “woozy”, “stressed”, “bored.”)

#### Social perception data analyses

Two 2 X 3 ANCOVAs (time by age group) were conducted with the sincere and insincere cue scores, again controlling for IQ. Matched t-tests of Time 1 and Time 2 sincere and insincere cue detection scores assessed changes within each age group. Correct responses to “mean” or “not mean” judgments were summed for the sincere versus insincere vignettes (range = 0 to 3). Similar to cue detection scores, 2 X 3 ANCOVA’s identified differences in judgments scores between time points and age groups, and match t-tests assessed change over time within each age group.

Relative strengths or weaknesses in overall vignette performances were first assessed in a matched t-test between the sincere versus insincere cue detection scores. Then, two repeated-measure ANOVA’s identified relative strengths or weaknesses in participant’s abilities to accurately interpret the “not mean” or “mean” intention of protagonists within the three sincere and insincere vignettes, respectively.

Collapsing across age groups and time, Pearson correlations were conducted with the two cue detection scores and the combined total mean of these scores, with age, Composite IQs, the CBCL’s Attention Problem subdomain, the RBS-R Sameness Domain, and emotion recognition scores.

#### Relations between emotion recognition and social perception

A hierarchical multiple linear regression determined if emotion recognition predicted the detection of pertinent social cues, regardless of valence. As such, the dependent variable was the combined mean of sincere and insincere cue scores. Predictors in the first block included: age, Composite IQ, the CBCL Attention Problems and RBS-R Sameness/Rituals domain. Controlling for effects of these variables, the second block included the four emotion recognition scores.

#### Effect sizes

Effect sizes were calculated for all analyses. ANOVA effects sizes were estimated with partial eta squared, ηρ^2^, and the regression used R^2^ and ηρ^2^. For matched t-tests, Cohen’s *d* was calculated using the formula for paired samples.

## Results

### Preliminary analyses

ANOVAs determined if gender or growth hormone treatment status had a significant impact on emotion recognition and social perception scores, and would need to be controlled for in subsequent analyses. No significant differences emerged; see Tables A and B in S1 File. An ANOVA assessed differences in scores across PWS genetic subtypes. Consistent with previous literature, participants with Type I deletions had lower KBIT-2 Composite IQs (*M* = 61.53, *SD* = 14.94) than those with Type II deletions (*M* = 73.03, *SD* = 17.09) or mUPD (*M* = 73.56, *SD* = 17.66); *F*(188) = 5.96, *p* < .001. Controlling for IQ, no significant effects of genetic subtype were found in the social cognition scores; see Table C in S1 File. Finally, although the test-retest interval was not correlated with social cognition scores, we re-ran analyses with test-retest interval as a covariate. No effects of this time interval were found. Thus, none of the possible covariates (genetic subtype, gender, test-retest interval) were included in final analyses.

### Emotion recognition

#### Change over time

Table 2 summarizes mean scores for sad, fear, angry and happy at Times 1 and 2 for each age group, and the sample as a whole. Of the four emotions, only the 2 X 3 ANCOVA assessing fear proved significant; *F*(6,187) = 5.54, *p* < .001, *ηρ*^*2*^ = .16, with a significant main effect for time, F(1,187) = 16.63, p < .001, *ηρ*^*2*^ = .09. The sample as a whole improved in fear scores from Time 1 to Time 2.

**Table 2:** Mean scores and (standard deviations) for emotion recognition task across age groups of participants with PWS.

	5–10 years	11–19 years	> 20 years	Sample Total
**Sad Time 1**	2.55 (1.70)	2.07 (1.60)	1.78 (1.65)	2.20 (1.66)
**Sad Time 2**	2.66 (1.61)	2.27 (1.46)	2.07 (1.51)	2.33 (1.53)
**Sad Age Total**	2.60 (1.65)	2.17 (1.53)	1.95 (1.56)	2.26 (1.59)
**Fear Time 1**	1.46 (1.58)	2.03 (1.76)	1.21 (1.31)	1.58 (1.48)
**Fear Time 2**	2.76 (1.67)	2.73 (1.67)	2.55 (1.70)	2.49 (1.70)
**Fear Age Total**	2.02 (1.73)	2.05 (1.71)	2.00 (1.67)	2.03 (1.69)
**Angry Time 1**	3.00 (1.26)	2.48 (1.37)	3.00 (1.00)	2.79 (1.27)
**Angry Time 2**	3.00 (1.43)	3.16 (0.89)	3.04 (1.22)	3.08 (1.06)
**Angry Age Total**	3.00 (1.20)	2.81 (1.20)	3.02 (1.12)	2.93 (1.18)
**Happy Time 1**	3.80 (0.40)	3.75 (0.81)	3.84 (0.68)	3.79 (0.65)
**Happy Time 2**	3.85 (0.36)	3.94 (0.23)	3.91 (0.48)	3.91 (0.28)
**Happy Age Total**	3.82 (0.38)	3.84 (0.61)	3.89 (0.48)	3.85 (0.51)

Within age groups, matched t-tests of Time 1 versus Time 2 scores revealed significant differences in identifying fear in children, *t(*43) = -3.08, *p* = .004, *d* = .58, and adults, *t*(15) = -2.74, *p* = .016, *d* = .71. Although the adolescent group also increased in fear recognition over time, this change was relatively modest, with a small effect size, *t*(43) -2.24, *p* = .032, *d* = .19. Adolescents also showed an improvement in anger recognition scores, *t*(33) = -2.86, *p* = .007, *d* = .48. No significant changes were found within any age group in the recognition of sad or happy.

#### Relative strengths and weaknesses

A repeated measures ANOVA was conducted with the three negative emotions for the sample as a whole, collapsed across age groups and time (see Table 2 for means). Happy was not included as preliminary analyses indicated that these scores were significantly higher than all other emotions, with participants performing at near ceiling levels at both assessments. Mauchly’s Test indicated that the assumption of sphericity was met; *w* = .99; *X*^*2*^ (2) = 1.22. Significant differences emerged between the 3 negative emotions; *F*(1,186) = 39.61, *p*< .001; ηρ^2^ = .17. Bonferroni post-hoc comparisons revealed that participants had higher anger versus fear or sad scores, with no difference between fear or sad. Participants earned an average of 56% correct for sad, 50% correct for fear, and 73% for anger.

#### Correlates

Pearson correlations were conducted using data collapsed across both time and age group. Anger recognition was negatively associated with Attention Problems, *r*(187) = -.20, *p =* .011. KBIT-2 Composite IQs were correlated with recognition scores for fear, *r*(187) = .24, *p*< .001, and sad, *r(*187) = .23, *p*< .001.

#### Error analysis

A review of errors indicated that participant’s incorrect responses to negative emotions generally had a negative valence. Sad, for example, was misconstrued as anger in 53% of incorrect responses, and anger was misinterpreted as sad in 44.3%; see Table D in S1 File. Although the majority of errors identifying fear had a negative valence (73%), 11.4% were misconstrued as surprised, and 11.1% of errors had a positive valence (e.g., happy, courageous, proud). A mismatch of valence was also found in responses to sad faces (16.5% positive valence), but was relatively infrequent in anger (4.6%).

### Social perception

#### Change over time: Cue scores

Table 3 summarizes mean scores for sincere/benign and insincere/hostile cues for each age group at Times 1 and 2, and for the overall sample. The 2 X 3 ANCOVA’s were significant for both sincere/benign cues, *F*(6,187) = 12.32, *p* < .001, ηρ^2^ = .34, and insincere/negative cues, *F*(6,187) = 8.84, *p* < .001, *ηρ*^*2*^ = .27. For the sincere/benign cues, main effects were found for time, *F*(1,187) = 5.88, p = .017, *ηρ*^*2*^ = .04, and age group, *F*(2, 187) = 21.37, *p* < .001, *ηρ*^*2*^ = .23. On average, scores for the entire sample were higher at Time 2 than at Time 1. Bonferroni post-hocs indicated significant differences between all age groups, with children scoring lowest, and adults highest. For the insincere/negative cues, a significant effect for age-group was found, *F*(2,187) = 18.61, *p* < .001, *ηρ*^*2*^ = .27, with Bonferroni post-hocs revealing that, on average, children scored significantly lower than both adolescents and adults.

**Table 3:** Mean (SD) proportion scores of sincere/benign and insincere/hostile cues in social perception vignettes across age groups.

	5–10 years	11–19 years	> 20 years	Sample Total
Time 1 Sincere Cues	0.89 (0.69)	1.44 (1.17)	1.85 (1.20)	1.34 (1.08)
Time 2 Sincere Cues	1.61 (0.85)	1.61 (0.85)	2.15 (0.94)	1.81 (0.91
Age Total	1.21 (0.84)	1.52 (1.03)	2.00 (1.05)	1.55 (1.02)
Time 1 Insincere Cues	1.64 (1.11)	2.60 (1.20)	2.65 (1.42)	2.25 (1.30)
Time 2 Insincere Cues	2.26 (0.90)	2.64 (1.18)	2.91 (1.17)	2.62 (1.11)
Age Total	1.95 (1.06)	2.62 (1.18)	2.80 (1.27)	2.44 (1.22)

Within-age group, matched t-tests revealed that only the group of children made significant gains over time in their detection of both sincere/benign cues, t(43) = -3.15, p = .003, *d* = .81, and insincere/negative cues, t(43) = -2.35, p = .024, *d* = .54.

#### Change over time: Judgments

Table 4 presents mean scores for the correct “not mean” or “mean” judgments for the sincere/benign versus insincere/hostile vignettes (range = 0 to 3). As reflected in the total mean scores in Table 4, the ANCOVA assessing correct responses to the benign vignettes was not significant. In contrast, the ANCOVA of insincere/hostile vignettes was significant, *F*(6, 187) = 5.05, *p* < .001, *ηρ*^*2*^ = .18, with a main effect for age group, *F*(2, 187) = 8.56, *p* < .001, *ηρ*^*2*^ = .11, such that children scored lower than adolescents and adults. This main effect, however, was qualified by a significant age by time interaction, F(2,187) = 3.56, p = .031, *ηρ*^*2*^ = .05. Bonferroni post-hocs revealed that children scored lower than remaining groups at Time 1 only.

**Table 4:** Mean (SD) correct judgments of either “mean” or “not mean” to the sincere/benign and insincere/hostile vignettes across age groups.

	5–10 years	11–19 years	>20 years	Sample Total
Time 1 Sincere Judgments	1.77 (1.15)	1.52 (1.18)	1.72 (1.22)	1.66 (1.17)
Time 2 Sincere Judgments	1.47 (1.16)	1.17 (1.92)	1.70 (1.20)	1.46 (1.19)
Age Total	1.64 (1.15)	1.36 (1.19)	1.71 (1.20)	1.56 (1.18)
Time 1 Insincere Judgments	1.15 (1.13)	2.07 (0.99)	2.44 (0.70)	1.82 (1.13)
Time 2 Insincere Judgments	1.95 (1.10)	2.52 (0.74)	2.15 (1.08)	2.20 (1.01)
Age Total	1.52 (1.19)	2.27 (0.91)	2.27 (0.94)	2.02 (1.08)

Within age groups, matched t-tests revealed significant improvements in children’s judgments of mean intentions portrayed in the insincere/hostile vignettes, *t* (43) = -2.87, *p* = .007, *d* = .60, but in no other age group. Exploring this finding, follow-up t-tests revealed that children made significant gains in all three of the insincere /hostile vignettes.

#### Relative strengths and weaknesses

Robust strengths were found in the detection of insincere/hostile cues relative to sincere/benign cues, *t*(93) = -14.83, *p* < .001, *d* = 1.75. Participants were also more likely to make the correct “mean” judgments in the insincere/hostile vignettes compared to the correct “not mean” conclusion in the sincere/benign vignettes, *t*(93) = 3.05, *p* = .003, *d* = .41. A within-group, repeated measure ANOVA determined if participants were better at judging the intent of story protagonists in any one of the three sincere/benign vignettes. Mauchley’s test indicated that the assumption of sphericity was met; *w* = .96, *X*^*2*^ (2) = 1.19. A significant effect was found, *F*(2,374) = 20.71, p< .001, ηρ^2^ = .17. Bonferonni post-hoc analyses indicated that participants had relative strengths in judging the emotional excuse scenario, and weaknesses in the subtle apology vignette. The within-subjects repeated measure ANOVA assessing the three insincere/hostile vignettes was not significant, *F*(2, 374) = 1.72, *p* = .18, indicating relatively even performances across these scenarios.

#### Correlates

Collapsing across test times and ages, Table 5 summarizes Pearson correlations of sincere/benign and insincere/hostile cue scores, and the total cue score, with age, K-BIT2 Composite IQ, CBCL Attention Problems, RBS-R Sameness/Rituals, and the four emotion recognition scores. Inattention was negatively associated with cue detection, while IQ and recognition of sadness, fear and happy were positively correlated with these scores. Correlations were not significant between vignette judgment scores and IQ, attention problems, and emotion recognition.

**Table 5:** Pearson correlations between social perception cue scores and behavioral and emotion recognition scores.

Correlates	Sincere/Benign Cues	Insincere/Hostile Cues	Total Cues
Age	.19*	.14	.17*
Composite IQ	.28***	.19**	.25**
Attention Problems	-.28***	-.24**	-.26**
Sameness/Rituals	-.09	-.13	-.11
Fear	.26***	.20**	.24**
Sad	.28***	.23**	.26**
Angry	-.08	-.09	-.09
Happy	.23**	.28***	.27**

Notes

*p< .05

**p< .01

***p < .001.

### Predictors of social perception

The hierarchical multiple linear regression identified the extent to which participant’s abilities to recognize emotions facilitated their detection of social cues in general, regardless of valence. Two factors led to the decision to use total cue scores. First, the pattern of correlations in Table 5 was remarkably similar for the two cue valance scores, and the total cue score. Second, even though relative strengths were found in detecting negative cues, there is no theoretical justification for the idea that *predictors* of cue detection will vary by cue valence. Without a framework for interpreting the results, the total score offered a parsimonious solution that addressed our hypothesis that emotion recognition, or other variables, would predict the detection of pertinent cues.

The dependent variable was the mean of the benign and hostile proportion scores, collapsed over time and age groups. Predictors in the first model included four control variables that could detract from, or facilitate, cue detection: age, K-BIT2 IQ, and the CBCL Attention Problems and RBS-R Sameness/Rituals subdomains. Controlling for these variables, the second model added sad, fear, happy and angry recognition scores. We ensured that assumptions were met regarding linearity, collinearity, outliers, and normality of data.

The first model was significant, *F*(4,184) = 7.23, *p* < .001, *R*^*2*^ = .16, with effects emerging for age, IQ and Attention Problems. The second model was also significant, *ΔF*(8,184) = 6.55, *p*< .001, *ΔR*^*2*^ = .13, with additional effects noted for recognition of happy and sad. Overall, the model accounted for 29% of total variance in social cue scores; results for each model are summarized in Table 6. As indexed by standardized β’s, recognition of happy was the strongest predictor, followed by age, sadness recognition, IQ, and attention problems.

**Table 6:** Results of regression analysis predicting total social perception scores.

	*B*	*SE B*	β	*t*	ηρ^2^
**Model 1**					
Age	.040	.016	.208	2.56**	.038
IQ	.035	.010	.290	3.61**	.078
Attention Problems	-.274	.129	-.170	-2.09*	.029
Sameness/Rituals	-.189	.320	-.045	-0.59	.002
**Model 2**					
Age	.041	.015	.217	2.67**	.049
IQ	.024	.009	.197	2.53**	.043
Attention Problems	-.252	.122	-.160	-2.06*	.030
Sameness/Rituals	-.251	.301	-.060	-0.84	.005
Happy	.939	.290	.233	3.75**	.066
Sad	.274	.098	.210	2.75**	.048
Angry	-.206	.124	-.117	-1.66	.018
Fear	.096	.093	.079	1.03	.007

Notes

*** p< .001

** p< .01

* p< .05.

## Discussion

Successfully navigating the social world depends on recognizing emotions and using social cues to determine the intentions of other people. Despite some gains over time, people with PWS have deficits in both of these critical areas of social cognition. Findings have novel implications for interventions to help people with PWS strengthen or acquire specific social cognition skills.

Participants readily identified happy, and they were significantly better at identifying anger than sadness or fear, a pattern also seen in a previous study [16]. Over time, all three age groups made significant gains in their recognition of fear. With the exception of adolescents, these gains reflected medium to large effects. Adolescents also improved in their recognition of anger. In general, however, performances were still relatively poor, with participants earning an average of 50% and 56% correct for fear and sad, respectively. No significant gains were seen in the recognition of sad, and sad was often mistaken for anger, and anger for sad.

Typically developing children reliably recognize basic human emotions from facial expressions (specifically, happy, sad, angry) by 4 to 6 years of age [24,25]. In a large, normative sample of children, rates of identifying happy, sad and anger in 6 year olds did not differ from 16 year olds. Fear recognition, however, increased gradually in this cohort such that 9 to 10 year olds were approximately 50% accurate, and 16 year olds, 76% accurate [24]. Thus, fear and other complex or more nuanced emotions (e.g., disgust, contempt, surprised) continue to evolve in adolescence and young adulthood [24, 41].

Compared to these normative trajectories, those with PWS were strikingly off. Older participants did not perform better than younger ones, and within age groups, improvements over time in fear and anger essentially brought the three age groups to the same level of performance. Participants achieved a certain level of competency, but advancing age did not necessarily lead to higher scores. Unlike the general population, then, people with PWS appear to have an atypical developmental trajectory in emotion recognition.

The errors made by participants further implicate an altered developmental trajectory of emotion recognition in PWS. When they were incorrect, participant’s often confused sadness and anger, and fear with surprised or other negative emotions. Widen [42] proposes that young children initially use valence to differentiate within two broad emotion recognition categories: “feels good” versus “feels bad.” Toddlers and young children start by identifying one emotion, happy. They then recognize either sad or angry, and by age 4, they can differentiate sad from anger. Children subsequently recognize either fear or surprised, and over time, differentiate between these two. Participant’s incorrect responses to angry, sad or fear generally had a negative valence or overtone (e.g., “stressed out”, “bored”, see Table D in S1 File), reflecting the “feels bad” category. As such, they could make good use of valence, but their confusion between sad and angry reflect difficulties at the earliest stages of emotion recognition development.

Consistent with previous work [16], KBIT2 Composite IQ’s were significantly correlated with emotion recognition scores for fear and sad. Thus, the cognitive resources of individuals with relatively high IQ’s may have enabled more accurate scores. General cognitive ability also plays an important role in emotion recognition in typically developing children and adolescents [24], and in other genetic, neurodevelopmental disabilities, including Williams syndrome and Down syndrome [43]. Martínez-Castilla and colleagues [43] concluded that impaired cognitive functioning in individuals with both of these syndromes constrained their emotion recognition performance, which advanced to a certain level, then became static did not get better with advancing age.

Future studies are needed to establish how emotion recognition deficits in people with PWS impact their social exchanges with others. Successful social interactions require abilities to detect and respond to shifts in the mood states of other people, or to share affect with them. Being emotionally aware of, or in tune with, the affect of others is instrumental in establishing close, reciprocal relationships [44]. A reasonable prediction for further study is that emotion recognition impairments in people with PWS are associated with their generally poor peer relationships [9].

Several themes emerged from participant’s responses to the social perception tasks. First, the sample as a whole improved over time in their ability to detect pertinent social cues, regardless of valence. Between age group findings point to a developmental progression in the detection of sincere/benign cues, with significant increments in scores between children, adolescents and adults. Increases were more substantial for the sincere cues, in part because of the significant, meaningful strength in participants’ detection of negative or hostile social cues. In addition to their relative strength in observing hostile or negative cues, participants were also more likely to correctly interpret the “mean” intent of protagonists in the insincere/hostile (versus sincere/benign) vignettes. Participants thus performed relatively well detecting peer rejection in the context of trickery, lying, and ridicule.

In contrast, participants performed poorly in judging the sincere intentions of others in the vignettes depicting accidental mishaps. Although participant’s noticed more sincere cues over time, they did not necessarily use these cues to form increasingly accurate interpretations of these vignettes. No significant improvements over time were found in participant’s interpretations of the sincere vignettes, as assessed both within and between age groups.

Even so, meaningful differences emerged across the sincere/benign vignettes that partially support the hypothesis that more salient cues would facilitate performance. Consistent with our prediction, participants performed poorly on the paper-scribbling vignette, with its’ subtle “uh-oh” embarrassed apology. Surprisingly, however, most did not take advantage of a clear, blatant cue (“I’m so sorry. I should have been watching where I was going”) in order to draw the correct “not mean” conclusion in the dropped papers vignette. Unexpectedly, they performed significantly better in the vignette that lacked a clear apology but instead included a strong emotional exchange. Perhaps, then, affect salience facilitated participants’ correct perceptions of this social situation.

Why, then, did most participants make the wrong call when judging sincere intentions of others, and the correct call in perceiving hostile intentions? Several explanations seem plausible. First, social interactions present complex, divergent stimuli that compete for attention, and social cues that arouse and dominate are more likely to remembered [45]. The gravity of the negative events won out (e.g., anger of the girl with dropped papers), and outweighed participant’s perceptions or recall of opposing or lower-priority cues (e.g., apology from the person who bumped into her).

The salience of negative events is also reflected in predictors of social cues. As expected, emotion recognition skills, specifically for happy and sad, were significant predictors of participant’s detection of cues, accounting for 11.4% of variance. Surprisingly, however, emotion recognition abilities were not associated with accuracy in judging the “mean” or “not mean” intentions of protagonists. Thus, even when participants had emotion recognition cues to employ to their benefit, they still could not move beyond the negative event itself to correctly interpret the sincere intentions of others.

Second, cognitive and attentional processes are recruited in perceiving social cues; one must attend to cues in order to encode and interpret them. Both IQ and attention problems emerged as significant predictors of cue detection scores. Although cognition explained more variance in cue detection than inattention, attending to pertinent social cues is an essential step in accurately deciphering social interactions.

Third, participants’ relative strengths in detecting and judging hostile scenarios may relate to a phenotypic tendency to perceive the world though a negative lens. A “negative personality” has previously been noted in people with PWS, primarily as a shorthanded way to capture such problems as irritability, argumentativeness, inflexibility, and insistence upon sameness [31]. Moving beyond such behaviors, Key et al., [46] used event-related potentials to assess neural responses of 24 adolescents and young adults with PWS to social and nonsocial stimuli that had both positive and negative emotional valences. Larger anterior late positive potential amplitudes were found for negative (versus positive) nonsocial stimuli and facial expressions at right fronto-temporal locations. Results suggest a bias toward negative emotional expressions (e.g., angry face) and nonsocial stimuli (e.g., mean looking dog).

It is important to emphasize, however, that people in general have a negativity bias that includes, among other factors, greater attention and cognitive processing of negative stimuli, and ascribing more complexity to negative stimuli [47]. Unlike the general population, however, a negativity bias in people with PWS may be especially problematic as the syndrome’s phenotype includes proneness for such features as irritability, argumentativeness, and inflexibility.

Although this is the first study to longitudinally examine both social perception and emotion recognition in PWS, several study limitations deserve mention. First, although the emotion recognition task in this study is standardized and widely used, it is static in nature. Skwerer and colleagues [48] administered both static emotion recognition (restricted to the eye region) and dynamic facial expressions (short video clips) to individuals with Williams syndrome and others with intellectual disabilities. Relative to the static eyes, both groups performed better with dynamic facial stimuli. Moving faces are ecologically valid, yet people with PWS, like those with other developmental disorders, may be slow in recognizing affect from dynamic stimuli [49]. If so, they could be disadvantaged in processing facial expressions as they occur in vivo, especially in complex or fast-paced social interactions.

In a related weakness, the study did not assess the many cues, aside from emotion recognition, that people use to interpret social situations. Extending beyond the spoken word, such cues include nonverbal body language, and such acoustic properties of speech as tone of voice, intonation, pitch or prosody [3]. Participants with PWS were more apt to accurately interpret the “not mean” intention of the protagonist in the scenario involving a loud and emphatic emotional exchange. Perhaps, then, future studies could explore how people with PWS use the vocal properties of speech, as well as nonverbal cues, to guide their social perceptions.

Third, we administered a social perception task that has not been widely used, and with unknown test-retest reliability. Even so, given the two-year average lag time between assessments, it is unlikely that results are attributable to practice effects. Although other social perception tasks and questionnaires exist, they were primarily designed for patients with schizophrenia or other psychiatric disorders [50]. Given our study sample, we instead opted to use a measure with a sound theoretical basis that was specifically developed for individuals with intellectual disabilities.

An additional weakness is that the study used a measure that captures the behavioral manifestations of inflexibility in PWS, but is not a sensitive index of cognitive inflexibility. The RBS-R Sameness/Rituals domain was not associated with social cognition performance, yet cognitive flexibility is crucial for accurate social perceptions and interactions [18]. Future social cognition studies might thus administer tasks that tap elements of cognitive switching that are known to be impaired in PWS, including engaging and disengaging attention, response inhibition, and task set reconfiguration [19]. Until such studies are performed cognitive inflexibility should not be ruled out as a possible contributor to social cognition deficits in PWS.

Finally, the study did not identify differences in social cognition in PWS relative to people with intellectual disabilities in general, or with other genetic syndromes. In this vein, Leffert et al. [34] also found that children with intellectual disabilities had trouble getting beyond the salience of negative events to make correct judgments of vignettes. It thus remains unknown if emotional recognition or social perception findings are distinctive to PWS. Although future work might include comparison groups, findings nevertheless have important implications for interventions in PWS.

Social cognition and social skills interventions abound in other disorders, especially autism spectrum disorder and schizophrenia. Recent social cognition intervention advances in these disorders include: integrating modeling from typical peers in theater productions [51]; creating diverse virtual reality training platforms [52]; using robotics to teach specific skills [53]; simultaneously treating multiple social cognitive domains [54]; using brain imaging as a biomarker for response to social cognitive treatment [55]; and implementing multi-modal trials that combine medication with social training [56].

In contrast, no studies have been published on the effectiveness of *any* type of formal social training or intervention program in PWS. The current study, however, points to specific targets for future interventions. The detection of pertinent social cues may improve with age, but the accurate interpretation of them does not. Interventions might thus focus on how to understand social cues, including basic emotions, and translate them into accurate judgments of social exchanges. Areas of relative strength (i.e., recognition of happy, angry, interpreting insincere/hostile cues) could be used as a template for addressing weaknesses in judging sincere intentions. Throughout, the focus should be on using social cues as evidence that can alter faulty thinking that leads to misperceptions of social interactions [57].

People with PWS have historically been described as “egocentric” [58], yet all people are egocentric. We must deliberately jettison our own perceptions in order to see the world through the eyes of others. Although these processes unfold in the typical population with learning and maturation, innovative interventions are increasingly and successfully used in patient populations with deficits in social cognition, including those with neurodevelopmental disabilities.

## Supporting information

S1 File **Table A**. Mean scores and (standard deviations) for emotion recognition and social perception tasks across gender. **Table B**. Mean scores and (standard deviations) for emotion recognition and social perception tasks across participants on growth hormone treatment (GHT) (n = 62) versus treatment naïve participants (n = 32). **Table C.** Mean scores and (standard deviations) for emotion recognition and social perception tasks across genetic subtypes of PWS. **Table D.** Types of participant errors in emotion recognition, collapsed across time and age groups.
(DOCX)
