# Supplementary material for: Profiles and trajectories of impaired social cognition in people with Prader-Willi syndrome
Source: PLoS One. 2019 Oct 17;14(10):e0223162. doi: 10.1371/journal.pone.0223162 (PMC6797185; doi:10.1371/journal.pone.0223162)
Supplement: S1 File — Table A. Mean scores and (standard deviations) for emotion recognition and social perception tasks across gender. Table B. Mean scores and (standard deviations) for emotion recognition and social perception tasks across participants on growth hormone treatment (GHT) (n = 62) versus treatment naïve participants (n = 32). Table C. Mean scores and (standard deviations) for emotion recognition and social perception tasks across genetic subtypes of PWS. Table D. Types of participant errors in emotion recognition, collapsed across time and age groups. (DOCX) [file pone.0223162.s001.docx]

**SI. Supporting Information**

**Table A**. Mean scores and (standard deviations) for emotion recognition and social perception tasks across gender.

| **Emotion Recognition** | **Males** | **Females** |
| --- | --- | --- |
| Fear Time 1 | 1.74 (1.60) | 1.41 (1.55) |
| Fear Time 2 | 2.91 (1.49) | 2.03 (1.82) |
| Sad Time 1 | 2.25 (1.67) | 2.06 (1.67) |
| Sad Time 2 | 2.46 (1.50) | 2.20 (1.54) |
| Angry Time 1 | 2.97 (1.18) | 2.64 (1.33) |
| Angry Time 2 | 3.23 (0.92) | 3.02 (1.16) |
| **Social Perception** | **Males** | **Females** |
| Sincere/Benign Time 1 | 1.39 (1.21) | 1.36 (0.95) |
| Sincere/Benign Time 2 | 1.78 (0.76) | 1.77 (1.07) |
| Insincere/Hostile Time 1 | 2.32 (1.39) | 2.31 (1.16) |
| Insincere/Hostile Time 2 | 2.68 (0.90) | 2.62 (1.23) |

**Note**: No significant differences were found between males versus females.

**Table B.** Mean scores and (standard deviations) for emotion recognition and social perception tasks across participants on growth hormone treatment (GHT) (n=62) versus treatment naïve participants (n=32).

| **Emotion Recognition** | **Off GHT** | **On GHT** |
| --- | --- | --- |
| Fear Time 1 | 1.23 (1.40) | 1.59 (1.63) |
| Fear Time 2 | 2.25 (1.73) | 2.56 (1.71) |
| Sad Time 1 | 1.80 (1.71) | 2.33 (1.64) |
| Sad Time 2 | 2.00 (1.41) | 2.43 (1.54) |
| Angry Time 1 | 2.93 (1.08) | 2.71 (1.36) |
| Angry Time 2 | 3.17 (1.16) | 3.09 (1.03) |
| **Social Perception** | **Off GHT** | **On GHT** |
| Sincere/Benign Time 1 | 1.49 (1.15) | 1.32 (0.90) |
| Sincere/Benign Time 2 | 1.78 (0.90) | 1.96 (0.93) |
| Insincere/Hostile Time 1 | 2.32 (1.27) | 2.32 (1.30) |
| Insincere/Hostile Time 2 | 2.62 (1.04) | 2.86 (1.11) |

**Note:** No significant differences emerged between those on versus off GHT.

**Table C.** Mean scores and (standard deviations) for emotion recognition and social perception tasks across genetic subtypes of PWS.

|  | **Type 1 Deletion** | **Type II Deletion** | **mUPD** | | **Other^+^** |
| --- | --- | --- | --- | --- | --- |
| **Emotion Recognition** | | | | | |
| Fear Time 1 | 0.65 (0.98) | 1.71 (1.54) | | 2.07 (1.79) | 1.37 (1.68) |
| Fear Time 2 | 2.05 (1.70) | 2.08 (1.85) | | 3.03 (1.57) | 3.37 (0.51) |
| Sad Time 1 | 1.50 (1.40) | 2.58 (1.62) | | 2.35 (1.59) | 1.00 (1.80) |
| Sad Time 2 | 1.90 (1.48) | 2.57 (1.62) | | 2.21 (1.57) | 2.50 (1.69) |
| Angry Time 1 | 2.89 (1.32) | 3.03 (0.99) | | 2.28 (1.48) | 3.25 (1.16) |
| Angry Time 2 | 2.74 (1.33) | 3.18 (1.05) | | 3.17 (1.02) | 3.50 (0.53) |
| **Social Perception** | | | | | |
| Sincere/Benign Time 1 | 0.86 (1.00) | 1.54 (1.07) | | 1.44 (1.09) | 1.35 (1.11) |
| Sincere/Benign Time 2 | 1.35 (1.03) | 1.92 (0.94) | | 1.76 (0.95) | 1.90 (0.76) |
| Insincere/Hostile Time 1 | 1.73 (1.18) | 2.40 (1.33) | | 2.44 (1.16) | 2.39 (1.29) |
| Insincere/Hostile Time 2 | 2.12 (1.14) | 2.77 (0.95) | | 2.77 (1.31) | 2.94 (0.76) |
| KBIT-2 Composite IQ | 61.53 (14.99) | 73.03 (17.04) | | 73.56 (17.66) | 66.89 (13.39) |

**S3 Table Notes:** **^+^** The two genetic subtypes represented in the Other group (Imprinting Mutations or Unique Deletions) groups had similar scores. Those with Type I deletions had lower IQs than participants with Type II deletions or mUPD. Controlling for IQ, no significant differences were found across genetic subtypes

**Table D.** Types of participant errors in emotion recognition, collapsed across time and age groups.

|  | **Expected, Correct Responses** | | |
| --- | --- | --- | --- |
| **Errors** | **Fear** | **Angry** | **Sad** |
| Sad | 18.2% | 44.3% |  |
| Angry | 26.8% |  | 52.9% |
| Other Negative^1^ | 32.1% | 27.5% | 25.7% |
| Positive^2^ | 11.4% | 4.6% | 16.5% |
| Surprised | 11.1% |  |  |

^1^ Examples of incorrect, negative responses include: Confused, out of it, shocked, weird, tired, strange, lonely, miserable, woozy, bored, grossed out.

^2^ Examples of incorrect, positive responses include: happy, excited, funny, silly, cheerful, content.
